# Digital age: The path choice of government-citizen value co-creation

**DOI:** 10.1016/j.heliyon.2024.e35482

**Published:** 2024-08-02

**Authors:** Jinguang Guo, Hanqi Zhang

**Affiliations:** School of Public Administration, Dongbei University of Finance and Economics, NO.217 Jianshan Street, Shahekou District, Dalian City, Liaoning Province, China

**Keywords:** Digital government, Citizen participation, Value co-creation, DART model

## Abstract

This study explores the relationship between government and citizen in the digital age and proposes a specific path to realize the value co-creation of government and citizen in the digital age. Quantitative analysis of 40 policy texts on “digital government construction” of 30 provincial and municipal governments in China before November 2022 was conducted using NVivo12. The case of Minsheng Cloud revealed that provincial and municipal government departments have not paid sufficient attention to citizen participation. Furthermore, citizens' participation ability and enthusiasm are not high. The study found that in the process of strengthening the construction of digital government, “government optimisation”, “citizen participation”, “governance guarantee”, “data governance” and “government digitization” are the key elements. We discuss the feasibility, necessity and coupling of the value co-creation theory to establish the benign interaction between government and citizens and realize co-creation. We find that the “DART” model can be co-created based on value, and that the continuous Improving the four aspects of “dialogue”, “access”, “transparency” and “risk assessment” will undoubtedly help realize the value co-creation of government and citizens in the digital era, and form a governance pattern of co-construction and sharing. It is therefore of great significance to promote the government's digital construction and digital governance capability to accelerate the construction of digital China and promote the modernisation of the national governance system and governance capability.

## 1 Introduction

Since the beginning of the 21st century, the new generation of information technologies such as artificial intelligence, big data, Internet of Things and Internet technology have brought profound impact on the development of human society. At the 19th National Congress, the concepts of “network power”, “digital China” and “smart society” were clearly put forward, and local government departments also responded positively.Judging from the excellent results of the leading pilot of government digital transformation, the ultimate result pursued by digital transformation is not only that the government can provide government services and improve social governance through information technology means, but also that digital technology can be used to change the original governance mode, fundamentally change the relationship between the government and citizens, and make citizens' participation in government governance become normal. Improve the service level and governance effectiveness of the government, optimize and upgrade the governance structure of the government (Chen and Liu, 2021.) [1], and achieve a governance pattern of co-construction and sharing.This requires not only the formation of a government-centered digital government, but also the establishment of a digital society centered on human connection, which is a key link to realize the connection between the virtual world and the real world. This means that there is an essential difference between digital government and e-government. If e-government is the Internet of government, that is, “government services + Internet”, then digital government can be said to be the Internet of government, that is, “Internet + government services” (Ma, 2021.) [2].It can be seen that the development of digital technology has brought about the need for changes in government governance styles. In this process of change, the relationship between citizens and the government has changed significantly. The first generation of e-government is characterized by “the realization of electronic transactions between citizens and enterprises and government agencies”, while the second generation of e-government focuses more on citizen participation in the delivery of e-government. Public (electronic) services. Finally, in the third generation of e-government, citizens are seen as potential sources of data and co-creators of public services (D and Lin, 2023.) [3].Therefore, how to stimulate citizens' subjectivity, improve citizens' participation, enable the government and citizens to form a synergistic symbiosis and value co-creation relationship, provide citizens with accurate and personalized services, and improve citizens' satisfaction and happiness have become the goals pursued by the construction of digital government.

China's digital government transformation is underway, and it's clear that the participants involved in e-government development have diverse identities and interests. This diversity is driving the creation of value through “value co-creation.” The value co-creation of public services, especially digital public services, is a process in which citizens, as producers and consumers of digital public services, co-create with digital public service service organizations through various actions (Percy, 2005) [4]. Each stakeholder has the right or potential to create services of public value (Cerdan and Simon, 2022) [5]. This emphasizes that both parties must make full use of their experience, capabilities and resources in the service delivery process (Anssell and Torfing, 2021) [6].Its essence is to break the original unequal status between the government and citizens through the effective communication of information resources, and promote the transformation of government services to the direction of diversification and transparency. The subjectivity of citizens is more prominent, and individual needs are easier to be captured, so that the government and citizens can jointly build and share, and achieve value co-creation. Based on this, the overall trend of social development promotes the deepening and enrichment of interaction and co-creation between the government and citizens, so as to realize the effective connection of resources among various subjects (Vargo and Lusch, 2004.) [7], which also reflects the necessity of value co-creation between the government and citizens.

So in the context of digital technology, how do governments and citizens interact to achieve value co-creation? How to further promote citizen participation to achieve value co-creation? Firstly, this paper will start from the development process of government digital transformation, and sort out the development process of value co-creation between government and citizens in the digital era. Through the mining of relevant policy documents of “digital government construction” and the analysis of cases of “livelihood cloud” in recent years, the key elements of digital government construction are summarised. Then the coupling and feasibility of value co-creation applied to digital government service governance are analyzed. Finally, the “DART” model is used to build a basic path that can promote the value co-creation between the government and citizens.

## 2 Research and analysis of value co-creation in the digital age

### 2.1 Research on value co-creation

The theory of value co-creation first emerged in the field of economics in the 19th century. Norman and Ramirez proposed the idea of value co-production in their research, suggesting that value co-production is based on the interaction between producers and consumers and is dominated by enterprises with consumers participating in it. It also highlights the role of consumers in value creation, which provides a theoretical foundation for the production of value co-creation theory. Subsequently, different scholars have contributed to the further development of the concept and meaning of value co-creation theory, offering insights from various perspectives (Zeithaml et al., 1990). [8] believes that service providers and service users create value together through cooperation and interaction. (Prahalad and Ramaswamy, 2004) [9]proposed that value co-creation connects enterprises with spenders. Enterprises do not try to cater to consumers, but actively engage in dialogue with consumers and participate in the construction of service experience. (Adelek and Abdulrahman, 2011) [10] believes that value co-creation is a cooperative activity initiated by product and service providers in order to promote product and service innovation and make product and service providers and users obtain mutual benefits (Liu et al., 2011). believes that value co-creation is an active interaction between consumers and enterprises [11]. In this process, consumers actively contribute their own labor and wisdom, co-invent and co-design with enterprises, and jointly produce and provide valuable products, services and experiences for consumers. It can be seen that although different scholars have different ways to explain value co-creation, the core idea is that value co-creation is realized by the participants in the production and consumption of products or services through joint participation and interaction (Liu, 2016) [12].

Extensive research on value co-creation theory began after 2000, when Vargo and Lusch proposed the value co-creation theory of “service-oriented logic” (Vargo and Lusch, 2004) [13]. Prahalad and Ramaswamy proposed the value co-creation theory of consumer experience (Prahalad and Ramaswamy, 2004) [14]. Prahalad and Ramaswamy proposed the value co-creation theory in 2004. Based on this, two branches developed respectively: one is the value co-creation theory based on “consumer experience” proposed by Prahalad and Ramaswamy et al. (Vargo and Lusch, 2008) [15]. The other is the idea of value co-creation based on “service-oriented logic” proposed by Vargo and Lusch.

Consumer experience-led value co-creation means that customers and enterprises create value together in the cooperation and interaction of an equal relationship. In the process of mutual interaction and interactive creation of customer experience, enterprises and customers determine common goals, solve related problems, build personalized service experience, and improve service quality through interaction. In this process, customers participate as value co-creators, co-creators of experience and active actors to achieve value co-creation (Wang et al., 2014) [16].The DART model is proposed, which includes four dimensions: Dialogue, Access, Risk-benefits and Transparency. The “DART” model provides a basic framework for improving citizen participation and realizing value co-creation. In the process of value co-creation, the customer, as one of the producers, also becomes the risk bearer. With the rapid development of the Internet, enterprises have built a “platform” through the continuous empowerment of technology, which provides a direct channel for the realization of value co-creation to a certain extent, and creates opportunities for accurate identification of customer needs. Based on this, Ramaswamy proposed a new Value co-creation framework called Value Interaction Creation. He believed that all consumers could become participants in value co-creation. However, the final level of participation depends on the functional design and operational efficiency of the interactive platform (Ramaswamy and Ozcank, 2018) [17].

The service-oriented logic holds that service is the fundamental basis of all economic exchanges, and the process of value co-creation emphasizes the initiative of interaction between customers and enterprises. Vargo et al. also put forward eleven propositions of service-oriented logic successively, holding that different participants in the creation process are discoverers and integrators of relevant resources, and customers invest their own resources, knowledge, skills and experience into the production process, making them both producers and value creators, thus realizing value co-creation.

### 2.2 Research on the value co-creation of government services in the digital era

Especially in the context of the rapid development of digital technology and the Internet, the diversity and personalization of customer groups and needs make the role of “customer” gradually prominent, and the producer is no longer the center. Gradually focus on the value co-creation of service-oriented logic, enterprises participate in the value creation activities of customers through interaction with customers, and finally realize the value jointly created by both parties.

Value co-creation theory, regardless of its logic, emphasizes the importance of customers and effective interaction. Each stakeholder can effectively integrate the resources of all parties through effective interaction, and make reasonable allocation of resources to achieve value co-creation. It can be seen that value co-creation is an effective way to integrate resources and avoid the failure of public services. It is inevitable that service failure is mainly determined by the intangibility, heterogeneity and synchrony of production and consumption of service itself.In 2016, Stephen proposed the concept of “public service failure” in his Research Agenda on Public Service Failure (Steven, 2016) [18]. He believed that the reasons for public service failure included two aspects: on the one hand, it failed to provide the services needed by citizens; On the other hand, the failure to provide services to citizens in line with established standards is essentially composed of service users, namely citizens' expectations of services and perceptions of processes (Stephen and Osborne, 2010) [19].The collection and utilization of information such as expectations and opinions require the government to pay more attention to citizens. Public service is no longer a one-way service supply but a multi-subject interaction, and it is no longer a one-way transfer of public resources but a process of co-creation of value between the government and citizens (Li, 2021) [20]. It can be seen that the process of truly realizing value co-creation is complex and requires the joint participation of multiple subjects, in which the government and citizens are the main participants. The core of value co-creation lies in the interaction between the government and citizens and the integration of social forces in the process of co-creation. However, based on the current social situation, shallow interaction can not achieve value co-creation, nor can it realize the integration of citizen behavior into government behavior, and the influence of citizen will on government behavior is weak.

Therefore, the research on value co-creation of government service has gradually developed in recent years. Compared with the enterprise field, value co-creation of government service is an innovative service tool. The essential demand of government service innovation is the transformation of the social technology system, and the ultimate manifestation is to realize the value co-creation of the upstream and downstream of the supply side and the value co-creation of the supply and demand side (Yu et al., 2021) [21].In particular, in the context of the continuous development and penetration of digital technology, the concept of value co-creation is used to give full play to the professional knowledge and skills of social groups, and the resources of the government and multiple social subjects are effectively used to realize the digital transformation of public services such as medical care, health, transportation and security, so as to provide citizens with better public services. Moreover, digital empowerment can alleviate the equalization of public services (Zhang and He, 2021) [22].The value co-creation of government services such as e-government, “Internet + government services” and digital governance is a new public service model for the government to improve the way of public services, create good interaction with the public and social subjects, and ultimately achieve a win-win situation. However, some scholars have clearly recognized that China's government service capacity is always in a situation of “strong in the east and weak in the west, strong in the south and weak in the north”, which indicates that improving the ability of government departments to carry out government affairs such as daily office work, public service and social governance in a digital and networked environment is a difficult problem that both academia and practitioners need to face (He and Yang, 2018) [23].

Scholars began to study the value co-creation in the field of government services in the 1970s. The research content focuses on the definition of value co-creation of government services (Yang et al., 2014) [24], the realization factors of value co-creation of government services and the role of value co-creation in improving the quality of e-government, and the obstacles faced by the public in the process of participating in value co-creation of government services (Subbiah&IbrhimI, 2011) [25]. And the role of social media in the value co-creation of government public services (Linders, 2012; Diaz-Diaz and Perez-Gonzalez, 2016) [26,27]. The concept of value co-creation is introduced into public service in order to realize the optimisation of public service through reasonable integration of resources. In this process, the participants mainly include two parts: on the one hand, government departments, namely public service providers, and on the other hand, citizens, social organizations, enterprises, etc. Some scholars divide public service into “rigid service” and “flexible service”, and believe that value co-creation of public service covers any public service process in which the public participates (Whitaker, 1980) [28]. Bovaird divides service value co-creation into nine models, consisting of the interaction of three public service planning alternatives and three public service delivery alternatives. And the following researchers further put forward four different forms of value co-creation of government services: joint planning, joint design, joint delivery and joint evaluation. Some scholars in our country have divided the “Internet + government service” value co-creation into two forms: First, the form of direct value co-creation, that is, the government and the public co-create service value; The other is indirect value co-creation, that is, all forms of co-creation interaction between government and enterprises, government and government, and government and the public with the purpose of serving users (Si and Hu, 2018) [29].

### 2.3 Research on the value co-creation of citizen participation in the digital era

Brudney and England proposed that in the process of public service value co-creation, five aspects should be taken into account: participation and feedback, initiative and negativity, cooperation and obedience, initiative and passivity, and individual and collective (Brudney and England, 1983) [30]. Liu Liu and Hu Guangwei, on the basis of technology acceptance theory and motivation theory, built a model of influencing factors of the public's willingness to participate in the value co-creation of e-government services. Among them, the public's willingness to participate is significantly affected by the degree of trust, participation motivation and platform acceptance. In the subsequent research, the interaction relationship among the elements of e-government service value co-creation strategy is more comprehensively understood through the PARTS model (Liu and Hu, 2015) [31].Based on the DART model, Addleke and Abdulrahman built an e-government service value co-creation process model composed of knowledge, skills, experience and interactive initiative (AdelekeI and Abdulrahman, 2011) [32]. Subbiah and Ibrahim also built a value co-creation framework for e-government services based on the DART model, which consists of three elements: individual unique value co-creation experience, citizen cooperation and government partners and competitors in acquiring management value and co-creating value (Subbiah and Ibrahim, 2011) [33]. (Nambisan, 2013) believes that citizens play four key roles in the value creation of public services, namely, explorer, advisor, designer and communicator [34] (Voorberg et al., 2013). found in their research that the roles of citizens in value co-creation can be divided into three categories: co-implementer, co-designer and co-initiator, among which loyalty, sense of responsibility, family composition and education of citizens are all important factors that determine whether citizens participate in value co-creation [35]. (Pang and Chen, 2016) found in the study that the public is both the recipient and the active publisher of information in public information services [36]. Effective participation and interaction of citizens can help improve service details, enhance citizens' sense of experience and service satisfaction, and help supervise government work and improve the quality of government services. The elderly are a particularly important part of the citizens and one of the users of digital public services. However, due to the limited acceptance and use of digital technology by the elderly themselves and the certain skewness of social resources, the popularization of digital age-appropriate degree is very important for the optimisation and improvement of public services. In addition, the development of digital-age-appropriate public services also requires multi-subject cooperation in value co-creation, which requires participants to reach a consensus on the concept of value co-creation, build a high-level applicable platform and establish corresponding policies and systems (Sun et al., 2023) [37].

## 3 'Digital government construction' policy text Measurement: characteristics and Deficiencies of citizen Variables

### 3.1 Selection of research samples

Grounded Theory was first proposed by American sociologists Bamey Glaser and Anselm Strauss in 1967. It refers to the continuous generalization based on the collection and decomposition of original data, from which concepts can be extracted and logic formed. It might be said that rooted theory has two important principles: one is “natural emergence” and the other is “continuous analysis and comparison”. It could be said that at the outset of the research, the researcher may only have a general research problem or area in mind, without any theoretical support. Over time, as the analysis of the data progresses, the conceptual categories may emerge naturally, which can then be used to construct a theoretical model. At the same time, it would be beneficial for the research to maintain a close connection between theory and data. The coding process is carefully coordinated and continuously refined. Through a process of continuous comparative analysis, links between conceptual categories are identified until a theory is developed.That is, the process of open coding, axis coding, core coding and saturation test is carried out, so as to rise to the theory.Digital government transformation is an emerging hot spot in recent years, and the research mainly focuses on e-government, government governance, government service and other hot spots in the process of digital government transformation. Policy text is an important carrier of China's policy thoughts and reflects the development plan of government construction in the next few years. Therefore, this paper will use the grounded theory method to analyze the policy text of digital government construction. However, due to the small number of policy documents related to the construction of digital government at the national level, this paper searches and selects the digital government implementation plans, guidance plans and other policy texts issued by provincial units through channels such as Peking University and provincial government portals for text analysis. By November 1, 2022, 40 documents related to digital government construction in 30 provinces have been selected and sorted out as research samples.

### 3.2 Open coding

In order to gain a deeper understanding of the theoretical models presented in the 40 policy documents, 30 policy texts were randomly selected for extraction. The remaining 10 policy texts were retained for theoretical saturation testing. In this paper, we have used Nvivo12 software to read through 30 policy documents in order to form a preliminary free node. We then proceeded to eliminate the initial concepts with repeatability and low frequency in the nodes, merge them and categorise them. Finally, 191 free nodes were extracted and summarised into 40 open codes (As shown in Table 1).

**Table 1:** Examples of open coding.

concept	Example policy document
Infrastructure	"Accelerate the construction of infrastructure System” (Ningxia)
Platform	"Improve the Intensive Government Cloud Platform” (Shandong)
Data operation	"Enhancing Digital Government Construction and Operation Capacity” (Guangdong)
Data security	"Improving Information Security Capability” (Gansu)
Data management	"Accelerate the improvement of government data resource system” follow the “one number of sources, multi-source check, dynamic update” (Guangxi)
Data opening	"Promote the full application of data in public services, social governance, macro decision-making, etc.” (Sichuan)
Data acquisition	"Accelerate Data Resource Collection and Aggregation” (Ningxia)
Digital economy	"Building an Innovation System for the Development of Digital Economy” (Hebei)
Business environment	"Implement the requirements of the Action Plan for Deepening the Comprehensive Reform of the Business Environment in our Province” (Guangdong)
Smart city construction	"Improve the New Smart City construction system and Mechanism” (Anhui)
People's livelihood service	"People's Livelihood Service Cloud Platform Group” (Jiangsu)
Government service	"Improving the functions of the Integrated Government Service Platform” (Henan)
Collaborative service	"New Mode of Government Cooperative Office” (Hubei)
Data decision	"Promote the in-depth mining and application of government big data to improve the government's decision-making and risk prevention capabilities” (Shandong)
Talent security	"Strengthening the Construction of talent Team” (Hubei)
Publicity and training	"Strengthening publicity and Training, Creating a Development Environment” (Yunnan)
Digital innovation	"Advance layout, integration and Innovation” (Guizhou)
Scene construction	"Promoting the Innovation of Government Data Application Scenarios” (Tianjin)
Digital ecology	"Building a Digital Ecological Collaboration Platform” (Shanghai)

### 3.3 Axial coding

As the focus of this paper is on the analysis of the relationship between government services and citizens during the transformation of digital government, based on the coding of open coding, further integration is carried out according to the requirements of spindle coding, and the focus is placed on government social governance and people's livelihood services, from which a number of concepts are extracted. For example, “service integration”, “self-service” and “sinking to the grassroots” are summarised as “government optimisation”, and “Internet + government” as “government service optimisation”, and “Internet + government” as “government service optimisation”. Internet + Government”, “Smart City”, “Digital Countryside”, etc. are summarised as “Digital Service and Governance”. The “Internet + Government”, “Smart City Construction”, “Digital Village”,etc. are categorised as “Digital Service and Governance" (As shown in Table 2).

**Table 2:** Axial coding.

Open coding	Axial Coding
Internet + government affairs, Smart city construction, Digital countryside	Digital services and governance
Quality foundation, Ability training, Information consumption power	Citizen participation
Service integration, Business coordination, Sinking grassroots, Optimisation window, Self-service, One Netcom office, One card, Online and offline integration, Consulting services, Process standardization, Convenience hotline, User portrait	Government affairs optimisation
Policy legislation, Organizational leadership, Talent security, Financial security, Supervision and efficiency, Technical ability, Publicity and training	Governance guarantee
Data acquisition, Data management, Data integration, Data development, Data sharing, Data operation, Data decision-making	Data governance
Government cloud platform, One network management, Government APP, Internet supervision, Pilot construction, Scene construction, Long-term mechanism	Government digitization

### 3.4 Data analysis

**Fig. 1:**
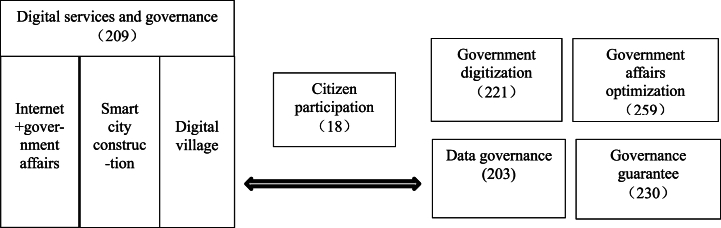
Components of livelihood services and governance.

This paper summarises the core elements of government livelihood services and governance construction during the digital government transformation period. The six elements are digital services and governance, citizen participation, government digitization, government affairs optimisation, data governance, and governance guarantee (As shown in Fig. 1). The numbers under each element in the figure are reference points obtained by coding. The analysis indicates that government affairs optimisation has the highest number of reference points. The focus during the digital government transformation stage is on upgrading and improving the original e-government construction, with an emphasis on optimizing the process of government affairs. Moreover, the scarcity of reference points for civic participation suggests inadequate consideration of this aspect in the ongoing digital government transformation process. Additionally, the reference points for ensuring governance and digitising government affairs are crucial in ensuring the provision of digital services in the development of digital government. Finally, the reference points for digital services and governance, and data governance are closely related. This suggests that digital services and governance, as well as digital technology capabilities, complement each other. The provision of digital services cannot be separated from the support of government digital governance capabilities.

### 3.5 Saturation test

If no new theoretical categories are produced in the analysis of the policy texts studied, then the theory can be considered to have reached saturation. In this study, 10 policy texts that were not involved in text analysis were used for coding and analysis, and no new categories and relationships were found from them, so it can be considered that theoretically saturation was reached.

### 3.6 Case studies

(1) Case Introduction

"People's Livelihood Cloud” is an interactive brand created by Shenzhen Guangming District Administration Bureau in the process of implementing “Internet + government services” and optimizing government services. With the help of advanced technologies such as “big data + cloud computing + artificial intelligence +5G″, “Livelihood Cloud” integrates the municipal 12345 government service hotline for the convenience of the people, the district mayor's hotline, the government website's message box, the People's Daily Online local leadership message board, “i Shenzhen” and other livelihood hotline channels. A work group network covering 33 functional departments, 6 streets, 31 communities, 8 state-owned enterprises and 4034 first-line grid workers, volunteers and YITF in the district has been built “horizontally to the edge and vertically to the end".Issued the “Guangming District Minsheng Cloud Management Measures (Trial)", established the Guangming District “Minsheng cloud” long-term operation mechanism; Compile and update the first edition of the Guangming District Civil Voice Matters Classification Standard, clarify the responsibilities of departments, and improve the accuracy of civil voice events allocation; Create the “intelligent learning + machine algorithm” of the growing intelligence distribution ability, the use of random forest classifier, through multiple decision trees for proof vote to determine the best solution, and finally based on intelligent learning, intelligent analysis capabilities, regularly push the people's livelihood hot information to the decision makers, focus on people's livelihood concerns, take the initiative to respond to social needs. Thus, the formation of party building leadership, science and technology empowerment, multi-network linkage, multi-layer coordination and multiple co-governance ensures that the problems reflected by the masses are managed online, handled offline, responded to everything, and have a pattern of dynamic and closed-loop management of the livelihood affairs of the Guangming District.

(2) Case analysis

By analyzing the case contents of “Livelihood Cloud”, the paper better interprets the core elements of government livelihood services and governance construction in the period of digital government transformation. The setting of “livelihood Cloud” makes information collection more efficient and shifts from “extensive” to “intelligent”. Realize the complementary advantages of online and offline, make the service more accurate, and transform from “separation” to “integration”; The creation of the platform has enhanced the interaction between the government and the people, and made the governance of grassroots social services more active, upgrading from “management” to “good governance”; Take the needs of citizens as the center, break through the information barriers, integrate data resources, and transform from the “edge” to the “center".The "People's Livelihood Cloud” interactive platform created by Guangming District of Shenzhen is a practical portrayal of improving government response, driving innovation in government governance, and mobilizing citizen participation, and it is also a powerful proof of the application of digital technology in government services to explore people's livelihood needs and social laws.

In conclusion, an analysis of the policy text and case studies reveals that the current digital government construction in China is primarily focused on innovation, optimisation and the supply of technical guarantee and service projects. However, it is crucial to acknowledge the importance of citizens in the process of digital government construction. Citizen participation represents a crucial link between government digital construction and digital service governance (as illustrated in Fig. 1). Citizens serve as both the producers of raw data and the recipients of government services, and their participation and feedback play a pivotal role in the optimisation of government services. The quality of the citizenry determines the extent of their participation, the ability to consume information determines the degree of participation, the ability to receive information determines the mode and channel of participation, and the ability to participate directly affects the success or failure of the transformation of the form of digital government governance. Secondly, the construction of digital platforms, the smoothness of interaction channels, the optimisation of the level of government services, the improvement of data governance capacity, and the governance guarantee are all important elements for the optimisation of digital services and governance. In order to further promote the construction of China's digital government and to facilitate the optimal utilization of digital technology in government services, it is essential to cultivate a productive interaction between citizens and the government. This interaction should be characterised by a spirit of value co-creation. In order to achieve this, it is necessary to gain a comprehensive understanding of the relationship between citizens and the government. This understanding should encompass the role of citizens and their potential. At the same time, it is vital to leverage digital technology to create a seamless platform for communication and feedback on government affairs. This will facilitate the integration of technology as a hub for intercommunication between the government and the citizen. The ultimate objective of value co-creation is to fully recognise the relationship between citizens and government, and to understand the role and potential of citizens.

## 4 Government-citizen path Options for value Co-creation

### 4.1 The coupling of value co-creation theory and digital government transformation

Based on the above research on value co-creation, value co-creation of government services in the digital era and value co-creation of citizens' participation in the digital era, and the summary of the construction of government livelihood services and governance in the current digital government transformation policy, it can be seen that: The activation of the vitality of citizen participation, with the help of emerging information technologies such as the Internet, enables citizens and the government to interact and effectively integrate the resources of multiple subjects to participate in government services and social governance under the common goal, enhance the sense of experience in the process of citizen participation, solve relevant problems together with participants, and meet the ever-rich multi-level and personalized needs of citizens. Improving the quality of government services and social governance is a key link that cannot be ignored, which is consistent with the ultimate goal pursued by the value co-creation theory. Based on the case analysis of "People's Livelihood Cloud”, it can be found that the smooth communication and feedback platform between the government and citizens and the ability of the government and citizens to jointly bear risks are the inaccessible components of value co-creation between the government and citizens in reality.

Therefore, based on the consumer-led value co-creation theory “DART” model, factors such as the relationship between value co-creation and participants, and the interaction between participants are coupled with the digital government transformation process (As shown in Table 3). This paper uses the “DART” model to study the problem of enhancing citizen participation and realizing value co-creation in the period of digital government transformation.

**Table 3:** Consumer-led value co-creation and the coupling of government-citizen relations in the era of digital government.

	Consumer-led value co-creation	Government-Citizen Relations in the Digital Age
Realize a goal	Co-creating economic value	Co-creation of value and improvement of service quality and governance capacity
value creator	Businesses and customers primarily, as well as other collaborators	Government and citizens as the main actors, as well as other participating actors
Co-creation of content	Realize value co-creation through multiple channels, multiple methods, resources, prices, service experience	Realization of co-creation through multiple participation channels, monitoring platforms, evaluation feedback, research and consultation
Mode of cooperation	One-to-one or one-to-many between the business and the customer, with both parties taking the initiative to launch	Government-citizen initiated, one-to-one or one-to-many, government-led
Relation-ship	Enterprises are the initiators, providing resources, platforms	Government as a leader, integrating resources and establishing platforms
	Customers are also producers and participate in value creation with their own experiences and demands.	Civic engagement in value creation for participants through feedback evaluation of service experiences and their own needs
driving force	Corporate pursuit of value, branding, reputation and competence	Purpose of the Government “to serve the people”, performance assessment, monitoring and evaluation
	The customer's pursuit of the use value of goods or the experience of services, the fulfillment of needs	Willingness of citizens to participate, sense of service experience, need fulfillment, psychological satisfaction
	information technology	information technology

**Fig. 2:**
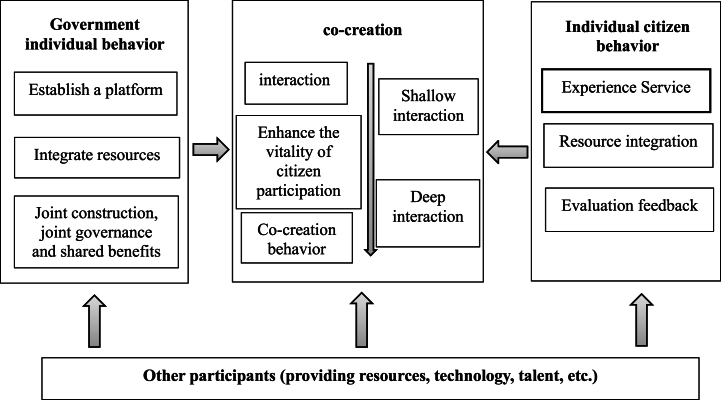
Process model of digital government and citizens to realize value co-creation.

The process of value co-creation is complex and requires the joint participation of multiple parties, with the government and citizens being the main participants. The process model of value co-creation (As shown in Fig. 2) highlights the interaction between the government and citizens as the core of value co-creation. However, in light of the current social situation, shallow interactions cannot achieve value co-creation, nor can they integrate citizens' behaviour into government behaviour. The influence of citizens' will on government behaviour is weak, which is also the crux of the current digital government transformation period. Service innovation and upgrading have not been able to truly meet the needs of citizens.

To achieve value co-creation, it is important to transition gradually from shallow to deep interaction. This process stimulates citizens' participation by enhancing their willingness and ability to participate, ultimately increasing the possibility of their involvement. Simultaneously, the government and citizens should continue to collaborate in interactive activities to establish shared goals. Citizens can then reintegrate their own resources. Feedback and expression of experiences and emotions form an equitable and positive interaction with the government. Under this consensus, the government can accurately understand the needs and aspirations of citizens. It can build a portrait of citizens' needs, overcome pain points, improve service forms, and enhance service quality. This will promote the digital government to truly achieve targeted, personalized services and the goal of serving thousands of people. This integration of citizen behavior into government behavior will create value and facilitate the process of value co-creation.

### 4.2 Meaning of the four elements of the DART model

**Fig. 3:**
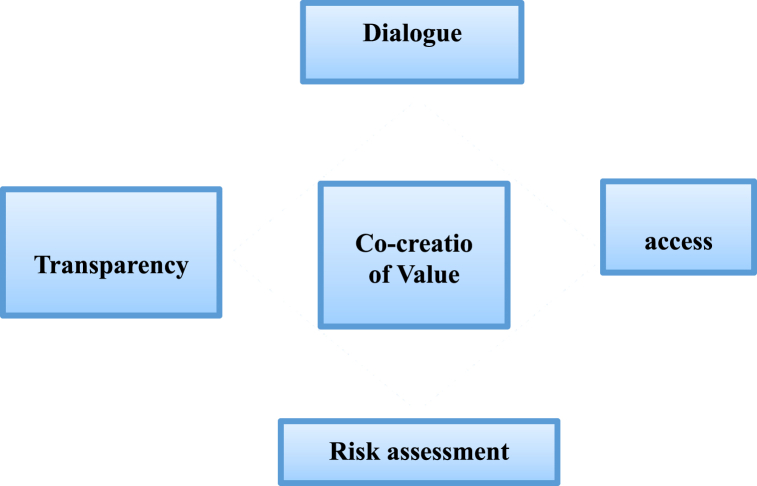
Value Co-creation DART model.

Source: Prahalad and Ramaswamy (2002).

The DART model offers a fundamental framework for enhancing citizen participation and achieving value co-creation. This paper aims to establish a path towards value co-creation by focusing on the four elements proposed in the model: dialogue, access, risk assessment, and transparency (As shown in Fig. 3).

#### 4.2.1 Dialogue

The essence of dialogue is the mutual communication between the parties involved in creating value. It requires an understanding and willingness to invest resources or energy in each other. In this stage of the process, the government and citizens communicate with each other on an equal footing. They seek to understand each other's needs and wishes, identify common goals and reach a consensus for cooperation. The aim is to integrate the values of the government and citizens, achieve reciprocity and share resources, information and knowledge. This iterative process forms a virtuous cycle that ensures smooth dialogue and symmetry of information between the co-creators, and prompts both sides to reach a higher level of willful compatibility. Thus, e-government is not only a means of modernizing internal operations, providing information, and delivering public services, but also an effective channel for citizens to participate in the democratic system and political process. It facilitates social exchange between individuals and the government, creating value for both parties (Alford, 2002; Ju et al.*,*2019*;* Bonsón et al., 2015.) [[38], [39], [40]]. Thus, governments and citizens are stakeholders in the value co-creation process, and the extent to which their benefits are captured influences their participatory behaviours and level of engagement (Wouters et al., 2021) [41].

#### 4.2.2 Access

Acquisition in the digital age differs from traditional acquisition, which involves ownership of items and exchange as a means of realizing value. However, in value co-creation, the concept of access differs from the traditional meaning and does not solely focus on ownership or experiential value that can only be provided by the company (Prahalad and Ramaswamy, 2002) [42]. Consumers are more interested in the experience rather than owning the product or service. This statement is in line with the government's responsibility to provide public services to citizens. Citizens have the right to use these services, but not necessarily to own them. When accessing services, citizens are more concerned with whether their needs are being met. It is the government's responsibility to provide effective access to services and information platforms, as well as ways for citizens to participate in the development and use of these services. This will ensure the quality of services and provide democratic legitimacy for the design and development of government services. Involving citizens in the development and use of government services ensures service quality and provides democratic legitimacy (Ju et al.,2019.) [43].

#### 4.2.3 Transparency

In the traditional relationship between firms and customers, firms may take advantage of information asymmetry and opacity of information about the production process to increase profits. Transparency of information can eliminate information asymmetry and build trust between organizations and individuals (Prahalad and Ramaswamy, 2004) [44]. The adoption of communication technologies can increase information dissemination, communication with stakeholders, and public input into government activities (Li and Feeney, 2014) [45]. When there is transparency of information between the government and individuals, it can lead to positive interactions and mutual trust between the parties involved. This, in turn, can result in better activation of citizens' participation and increased vitality of society as a whole. It enables citizens to reduce information asymmetry and identify inefficiencies, thereby changing the power dynamic between citizens and authorities (Katz and Halpern, 2013) [46]. At the same time, with the increasing amount of information available to citizens, transparency becomes more necessary.

#### 4.2.4 Risk assessment

Traditionally, in the context of transactions between firms and customers, risk refers to the possibility of damage to either party. It is often assumed that firms have better risk aversion than customers, and therefore tend to overlook risk in the transaction process. However, in the process of value co-creation, the customer, as one of the producers, also becomes a risk taker. When citizens participate in value co-creation, they also take responsibility as risk-takers in the government-citizen relationship. Therefore, in the process of value co-creation between the government and citizens, it is essential that both parties are aware of and clear about the risks and responsibilities they each bear. The government must adequately safeguard the interests and rights of citizens, including their ability to bear risks.

### 4.3 Constructing paths for Co-creation of value between government and citizens

#### 4.3.1 Improvement of dialogue mechanisms

Establishinga reliable dialogue mechanism is crucial for ensuring efficient communication and response between citizens and the government, which is essential for value co-creation.

To achieve this, we will establish multiple channels for dialogue. The interactive dialogue platform plays a significant role in promoting effective communication between the government and citizens. It strengthens information exchange, encourages citizens to express their demands and needs, enhances their participation enthusiasm, and improves the government's understanding of citizens' needs. With the support of information technology, the establishment of the dialogue platform should consider both online and offline forms. The offline platform provides opportunities and venues for direct face-to-face communication between citizens and government departments, making the dialogue and communication intuitive and in-depth. Simultaneously, the offline platform can facilitate communication between the government and citizens through various means, such as service experiences, roundtable discussions, service consultations, and hearings. The text is free from grammatical errors, spelling mistakes, and punctuation errors. No changes in content have been made, as per the instructions. This allows citizens to gain insight into policy information, relevant knowledge, development plans, and visions related to a specific service or governance project, and express their own thoughts, needs, and opinions. The language used is clear, objective, and value-neutral, with a formal register and precise word choice. The text adheres to conventional structure and formatting, with a logical flow of information and causal connections between statements. Online platforms can compensate for the limitations of offline platforms in terms of time, space, and speed of information transmission. By utilizing online consultations, network evaluation feedback, regular meetings, forums, and other forms of applications, information can be transmitted faster and more timely, even enabling real-time and effective communication. This allows the government to comprehensively and promptly understand the needs of citizens. The online platform serves as a test for the development of interactive government platforms, such as microblogs, portals, WeChat, and hotlines, to ensure their effective use.

Additionally, it enhances the government's ability to engage in dialogue with citizens by providing more channels for participation and expression in the digital age. This requires governments to be more responsive to dialogue and actively engage with citizens. On one hand, improving response ability requires enhancing the government's awareness of response, and on the other hand, improving the quality of work and response skills of relevant government personnel. Enhancing response consciousness directly reflects a change in government governance consciousness. It is necessary to break the traditional response mode of government and citizens and establish a “promising” and “service-oriented government”. The government needs to shift its focus from 'what should be done' to 'what can be done', adopting a more active approach to governance. Additionally, the digital government requires an improvement in staff quality and response skills, which is a new requirement for talent team construction. Civil servants must possess not only high quality, but also professional skills, a high professional level, a strong sense of responsibility, and service awareness. The ability to effectively communicate with citizens' questions and feedback, and skillfully solve and respond to their related needs is essential. It is important to use innovative response channels and methods, listen to the voices and opinions of the public in a timely manner, and strive to provide satisfactory solutions.

#### 4.3.2 Safeguarding access

Improving access channels can help citizens obtain services and information quickly, conveniently and in a timely manner, which is essential to promote equal interaction and foster positive dialogue.

Currently, citizens can acquire information through online, offline, and wireless channels. Online channels include government portal websites, government microblogs, government apps, government WeChat public accounts, and official marketing accounts on Douyin and short video platforms that have emerged in recent years. Wireless communication primarily involves transmitting and receiving information through TV broadcasts, telephones, mobile phones, SMS, and other means. It is important to note that this improved text adheres to the characteristics of objectivity, comprehensibility and logical structure, conventional structure, clear and objective language, format, formal register, structure, balance, precise word choice, and grammatical correctness.

In recent years, online channels, supported by network technology, have become widely accepted by the public compared to offline and wireless information channels, where no changes have been made to the content of the original text. Online channels offer benefits of speed, accessibility and transferring huge amounts of information over long distances. In addition, this channel lacks targeting. On the other hand, the offline approach offers flexibility and the ability to communicate face-to-face, making it suitable for all groups, especially those without internet access, the elderly, and technologically vulnerable groups. A disadvantage of the traditional approach is that it is expensive in terms of human and material resources, and has time and space limitations. On the other hand, the wireless approach has advantages such as controllable information reception, targeted audience, rapid transmission, ease of use and no time or space constraints, but the disadvantage is that the information transmitted is limited. Clearly, each approach has its advantages and disadvantages and is suitable for different groups. It is not enough to rely on a single access point to ensure citizens have access to information. This means communicating information in a way that takes into account the characteristics of the constituencies involved in obtaining information, and using multiple access points so that all stakeholders receive relevant information.

#### 4.3.3 Build a highly transparent information platform

High information transparency involves not only disclosing information but also ensuring its comprehensibility and reliability. The Chinese government has been committed to promoting transparency in government affairs as part of its digital transformation. The popularization of networking and information technology has made it necessary for government affairs information to be open, which tests the timeliness, accuracy, and ease of understanding of government information disclosure. It is also a requirement for the construction of a highly transparent information platform.

Firstly, the highly transparent information platform must ensure the timely disclosure of information. Although informatization has made it easier to transmit information, it has also raised the bar for the public's ability to distinguish between truth and falsehood. Therefore, ensuring that the public can obtain correct information in a timely and efficient manner can significantly reduce the spread of false public opinion and misinformation in society, while also enhancing the government's credibility. The timeliness of digital government data collection and operation is crucial. Additionally, the information platform should be highly transparent to ensure accurate and specific information disclosure. Government information should be consistent with reality, conveying the essence, beginning, and end of things comprehensively. This process requires the active cooperation and participation of relevant individuals. It also ensures the effective linkage of information resources among various government departments, which requires the establishment of a cross-departmental and cross-level government information network system. Finally, the high transparency information disclosure platform should ensure that the information disclosed is easy to understand. The government aims to provide information to all citizens in society, but there are differences in how different groups receive and understand this information. To ensure effective communication, it is important to innovate the way information is presented. In addition to traditional text-based formats, data visualization can be used to display information in a more intuitive and clear manner. At the same time, it can also consider the presentation of new media platforms such as Weibo, WeChat, and Douyin to effectively convey information and ensure full comprehension.Timeliness, accuracy and ease of understanding can improve information transparency, which can help citizens better understand government policies, services and governance orientation and participate in the process of value co-creation. To achieve digital government, technical level, talented team, financial security and internal organisational structure reform are required.

#### 4.3.4 Improvement of the safeguard mechanism

Both the government and citizens share responsibilities and risks in the process of value co-creation. To ensure a smooth and stable realization of this process, a sound guarantee mechanism is necessary. This mechanism should include information security, policy and law, and supervision guarantees.

Information security guarantee should be the first priority. Effective information transfer requires excellent information security technology to support it, preventing disclosure of citizens' personal information and narrowing the digital divide as much as possible. We must also build robust government information networks and basic citizen information bases, improve information security and confidentiality, and create a favorable digital information ecological environment. Secondly, we will enhance policy and legal safeguards. In order to maintain citizens' rights and interests during the process of value co-creation, it is necessary to introduce clear policies and laws that provide protection. It is essential to guarantee citizens' right to access and use information within the legal framework, as well as to protect their rights and interests when they are violated. This requires clear legal procedures and effective means of redress. These include defining what information requires disclosure, as well as its purpose, scope and use. By providing policy and legal guarantees, the status of both the government and citizens can be made more equal in the process of value co-creation. Additionally, a supervision mechanism should be established. The establishment of a supervision mechanism involves both citizen and government behaviour. It is necessary to create an 'Internet + supervision' platform that utilises both online and offline channels. The mechanism should cover every aspect of the value co-creation process and act as a constraint on co-creating subjects.

## Data availability

The datasets used in this study are available from theauthors on reasonable request.

## CRediT authorship contribution statement

**Jinguang Guo:** Project administration, Supervision, Writing – review & editing. **Hanqi Zhang:** Writing – review & editing, Writing – original draft, Methodology, Investigation, Conceptualization.

## Declaration of competing interest

The authors declare that they have no known competing financial interests or personal relationships that could have appeared to influence the work reported in this paper.
